# Association of Mandibular Morphological and Trabecular Bone Characteristics with Long-Term Outcomes Following First Permanent Molar Extraction: A Retrospective Study

**DOI:** 10.3390/diagnostics16203423

**Published:** 2026-10-21

**Authors:** Aybike Bas Ozturk, Gelengul Urvasizoglu, Rama Abdulkader, Nazrin Sariyeva, Fatih Sengul

**Affiliations:** 1 Department of Pediatric Dentistry, Faculty of Dentistry, Ataturk University, 25050 Erzurum, Türkiye; 2 Department of Oral and Maxillofacial Surgery, Faculty of Dentistry, Ataturk University, 25050 Erzurum, Türkiye

**Keywords:** first permanent molar extraction, second molar mesialization, spontaneous space closure, antegonial notch, mandibular morphology, fractal analysis, second premolar eruption, primary second molar, periapical index

## Abstract

**Background/Purpose:** This study aimed to evaluate mandibular morphological, trabecular, dental, and periapical factors associated with favorable long-term outcomes following early mandibular first permanent molar (FPM) extraction. **Methods:** This retrospective study included 60 patients with pre-extraction and ≥5-year follow-up panoramic radiographs. Patients were classified into favorable (FO; *n* = 30) and unfavorable (UFO; *n* = 30) outcome groups according to residual space and second permanent molar (SPM) axial position. Gonial angle (GA), antegonial notch depth (AND), notch angle (ANA), cortical thickness (ACT), and corpus and angulus fractal dimensions (FD) were assessed. Initial SPM and second premolar (SP)angulations, primary second molar (PSM)–SP status, and FPM periapical index (PAI) scores were also evaluated. Analyses included Bonferroni correction, exploratory multivariable logistic regression, and receiver operating characteristic (ROC) analysis. **Results:** AND was greater in the FO group (Bonferroni-adjusted *p* = 0.015) and remained associated with FO after adjustment (OR = 2.22 per millimeter; 95% CI: 1.19–4.15; *p* = 0.012). An extracted PSM with an unerupted SP was associated with higher odds of FO than a retained PSM (adjusted OR = 9.51; 95% CI: 1.45–62.50; *p* = 0.019). AND demonstrated moderate discrimination (AUC = 0.728; 95% CI: 0.600–0.856); a ≥2.0-mm threshold yielded 86.7% sensitivity and 53.3% specificity. Other morphological and fractal parameters did not differ significantly between groups; angulations and PAI were not significantly associated with outcome. **Conclusions:** AND and PSM–SP status were associated with FO. These exploratory findings require validation in larger, independent cohorts before clinical application, particularly given the wide confidence intervals for categorical estimates and the limited specificity of the proposed AND threshold.

## 1. Introduction

First permanent molars (FPMs) play an important role in dental arch development and the maintenance of occlusal stability; however, their prognosis may be compromised at an early age by dental caries, developmental enamel defects, and associated pulpal or periapical pathologies [1,2]. In FPMs with a poor long-term prognosis, extraction performed at an appropriate stage of dental development may allow the second permanent molar (SPM) to migrate mesially and spontaneously close the extraction space [1,3,4]. Nevertheless, spontaneous space closure is less predictable in the mandible than in the maxilla, and unfavorable outcomes (UFO), including residual spacing, mesial tipping, and rotation of the SPMs, may occur. A recent meta-analysis reported spontaneous space closure rates of 85.3% in the maxilla and 48.1% in the mandible [3].

Several dental and developmental factors that may influence spontaneous space closure following FPM extraction have been investigated. Chronological age, timing of extraction, the developmental stage and angulation of the SPM, and the presence of the third permanent molar (TPM) have been identified as potential prognostic factors [3,5]. Initial second premolar (SP) angulation and its position relative to the roots of the primary second molar (PSM) have also been evaluated, although their associations with successful closure remain inconsistent [4,6,7]. These findings support evaluating PSM presence and SP eruption status alongside initial tooth angulations. In addition, periapical pathology affecting the FPM may alter the local bone environment at the extraction site. Therefore, in the present study, pre-extraction FPM periapical index (PAI) scores were evaluated to explore whether radiographic periapical status was associated with subsequent spontaneous space closure.

The eruption pathway of posterior teeth and dentoalveolar relationships may be influenced by morphological variations associated with mandibular growth and remodeling [8]. Morphological features, such as the gonial angle (GA) and antegonial notch morphology, may serve as radiographic indicators of mandibular growth patterns and remodeling processes [9,10]. Accordingly, pre-extraction mandibular morphological characteristics may be associated with the mesial movement of the SPM into the extraction space and its subsequent long-term position. However, evidence regarding the relationship between mandibular morphological parameters and long-term outcomes following early FPM extraction remains limited.

The structural complexity and organization of trabecular bone can be quantitatively assessed on dental radiographs using fractal analysis (FA) [11,12]. Fractal dimension (FD), a quantitative measure derived from FA, has been used to characterize variations in trabecular bone patterns across various dental and skeletal conditions [12]. However, the potential association between pre-extraction trabecular bone architecture and long-term spontaneous space closure following early mandibular FPM extraction remains insufficiently characterized. Evaluating trabecular bone characteristics alongside mandibular morphological parameters may therefore provide further insight into the anatomical and structural differences associated with favorable outcomes (FO) and UFO.

This study aimed to evaluate a broad range of pre-extraction mandibular morphological, trabecular, dental, and periapical factors potentially associated with favorable long-term outcomes following early mandibular first permanent molar (FPM) extraction. The null hypothesis was that mandibular morphological parameters and trabecular bone characteristics would not differ significantly between the FO and UFO groups. An additional null hypothesis was that initial SPM and SP angulations, PSM–SP status, and FPM PAI scores would not be associated with long-term outcome.

## 2. Materials and Methods

This retrospective study was conducted using the archival records of patients who had received treatment and follow-up care at the Departments of Pediatric Dentistry and Oral and Maxillofacial Surgery, Faculty of Dentistry, Ataturk University. The study protocol was approved by the Ethics Committee of the Faculty of Dentistry, Ataturk University (Decision No. 55; date of approval: 21 August 2026).

### 2.1. Sample Size Calculation

An a priori power analysis was performed using G*Power software (version 3.1.9.7; Heinrich Heine University, Düsseldorf, Germany). The effect size was estimated based on a previous study investigating skeletal factors associated with spontaneous space closure following FPM extraction [4]. Based on the reported ANA values for the complete and incomplete space-closure groups (165.84 ± 4.63° and 162.00 ± 4.97°, respectively), an effect size (Cohen’s d) of approximately 0.80 was calculated. With a two-sided α level of 0.05, a statistical power of 80%, and an allocation ratio of 1:1, the minimum required sample size was calculated to be 52 patients (26 per group). A total of 60 patients (30 per group) were included in the present study.

### 2.2. Patient Selection

**Figure 1:**
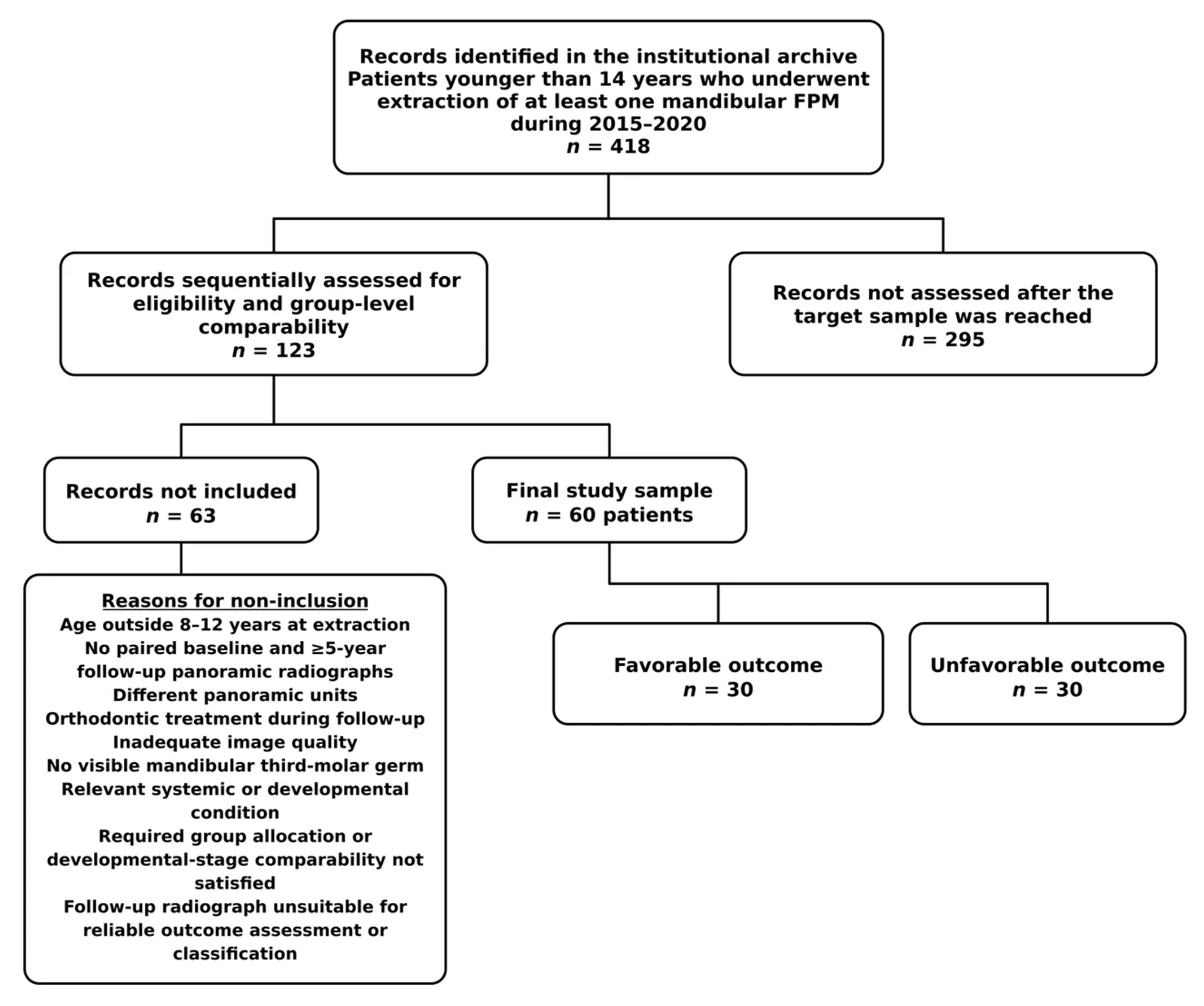
Flow diagram showing the retrospective record-screening and patient-selection process. Screening was terminated when the prespecified target sample of 60 patients, comprising 30 patients in each outcome group, was reached. FPM: first permanent molar.

The institutional archive contained 418 records of patients younger than 14 years who underwent extraction of at least one mandibular FPM between 2015 and 2020. Records were assessed sequentially against the predefined eligibility criteria until 30 eligible patients were included in each outcome group. Age at extraction and SPM developmental stage were considered during selection to achieve group-level balance rather than individual matching. Of 123 records assessed in detail, 63 were not included because of ineligibility or the group allocation and developmental balance requirements. The remaining 295 records were not assessed after the target sample was reached (Figure 1). The patient was the unit of analysis, with one mandibular extraction site evaluated per patient. In six patients whose contralateral mandibular FPM had been extracted at a different time, the side with better radiographic image quality was selected.

### 2.3. Outcome Classification and Dental Development

**Figure 2:**
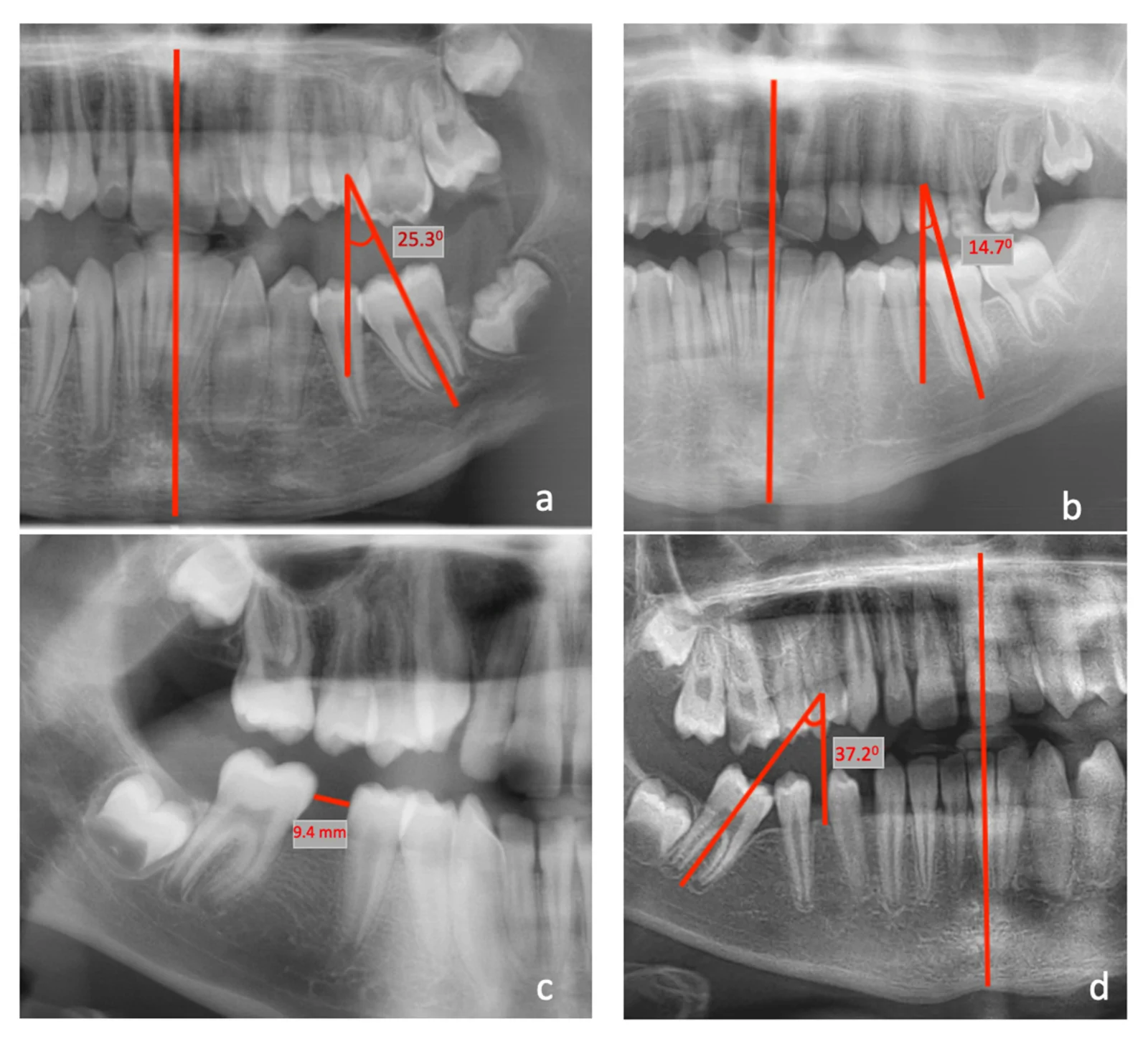
Representative examples of patient classification based on panoramic radiographs obtained at least 5 years after extraction. (**a**,**b**) Examples of patients classified into the favorable outcome (FO) group. (**c**,**d**) Examples of patients classified into the unfavorable outcome (UFO) group.

Outcomes were classified using panoramic radiographs obtained at least five years after extraction. Residual space between the SP and SPM and the SPM angle relative to the radiographic midline were assessed. The midline was defined by a line connecting the anterior nasal spine and the center of the mandibular symphysis. The FO group included 28 patients with complete space closure and an SPM–midline angle ≤ 30°, and two patients with residual space < 0.5 mm and angles of 17.4° and 22.7°. The UFO group included 13 patients with residual space > 2 mm and 17 patients with severe mesial tipping, defined as an SPM–midline angle > 30°; the smallest angle in the latter subgroup was 32.6°. Both spacing and angulation were considered because space closure alone could occur through excessive tipping (Figure 2).

To minimize the potential confounding effects of dental maturation and chronological age on mandibular morphology and trabecular bone structure, patients were selected to ensure comparability between the FO and UFO groups in terms of age and the developmental stage of the mandibular SPM. Demirjian stages A–H were coded as 1–8. A radiographically visible TPM germ preceding stage A was coded as 0. Developmental stages were analyzed as ordinal variables.

### 2.4. Imaging, Measurement Procedures, and Reliability

All baseline assessments were performed on pre-extraction panoramic radiographs obtained using the same unit (ProMax^®^, Planmeca Oy, Helsinki, Finland). Morphological measurements were performed using Turcasoft software (version 2026.3.27.1; Turcasoft Yazılım San. ve Tic. Ltd. Şti., Samsun, Türkiye). Linear measurements were recorded in millimeters using the imaging system’s calibration information; no additional correction for regional magnification or geometric distortion was applied.

Outcome classification, initial tooth angulations, PSM–SP status, and FPM PAI scores were assessed by one examiner (A.B.O.). Intraobserver reliability was assessed by repeating these evaluations in 10 patients after a one-week interval. For the repeated continuous dental measurements, the intraobserver ICC was 0.95.

The original morphological and fractal assessments were performed by two pediatric dentists (A.B.O. and F.S.) in consensus. Because outcome classification preceded these assessments, the examiners were aware of group allocation. For reliability assessment, 20 randomly selected baseline radiographs were anonymized and independently assessed by both examiners in two sessions two weeks apart. Image order was randomized for each session, and the examiners had no access to follow-up images, outcome classifications, or previous measurements. GA, AND, ANA, ACT, corpus FD, and angulus FD were reassessed. Intra- and interobserver reliability were evaluated using absolute-agreement intraclass correlation coefficients. ICC values for the morphological and fractal measurements ranged from 0.93 to 0.96.

### 2.5. Radiomorphometric and Dental Assessments

**Figure 3:**
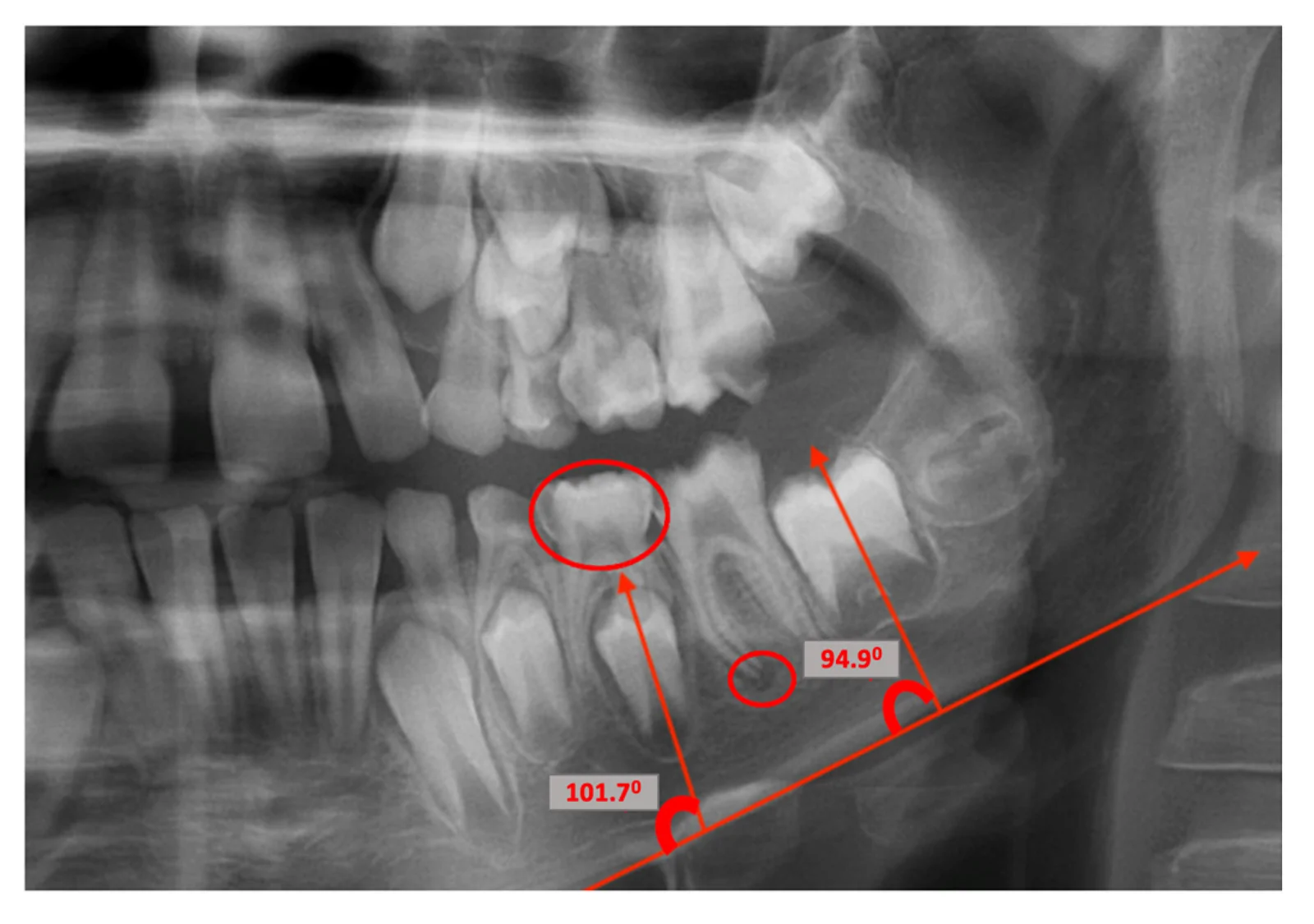
Evaluation of potential predictors.

Initial SPM and SP angulations were measured between their respective long axes and the mandibular plane. Baseline PSM–SP status was classified as: (1) PSM present; (2) PSM extracted with SP unerupted; or (3) SP erupted. FPM periapical status was assessed using the PAI, scored from 1 to 5, as a potential local factor associated with subsequent space closure (Figure 3). Extraction indications and the timing of maxillary or contralateral mandibular FPM extractions were inconsistently documented and were therefore not included as study variables.

**Figure 4:**
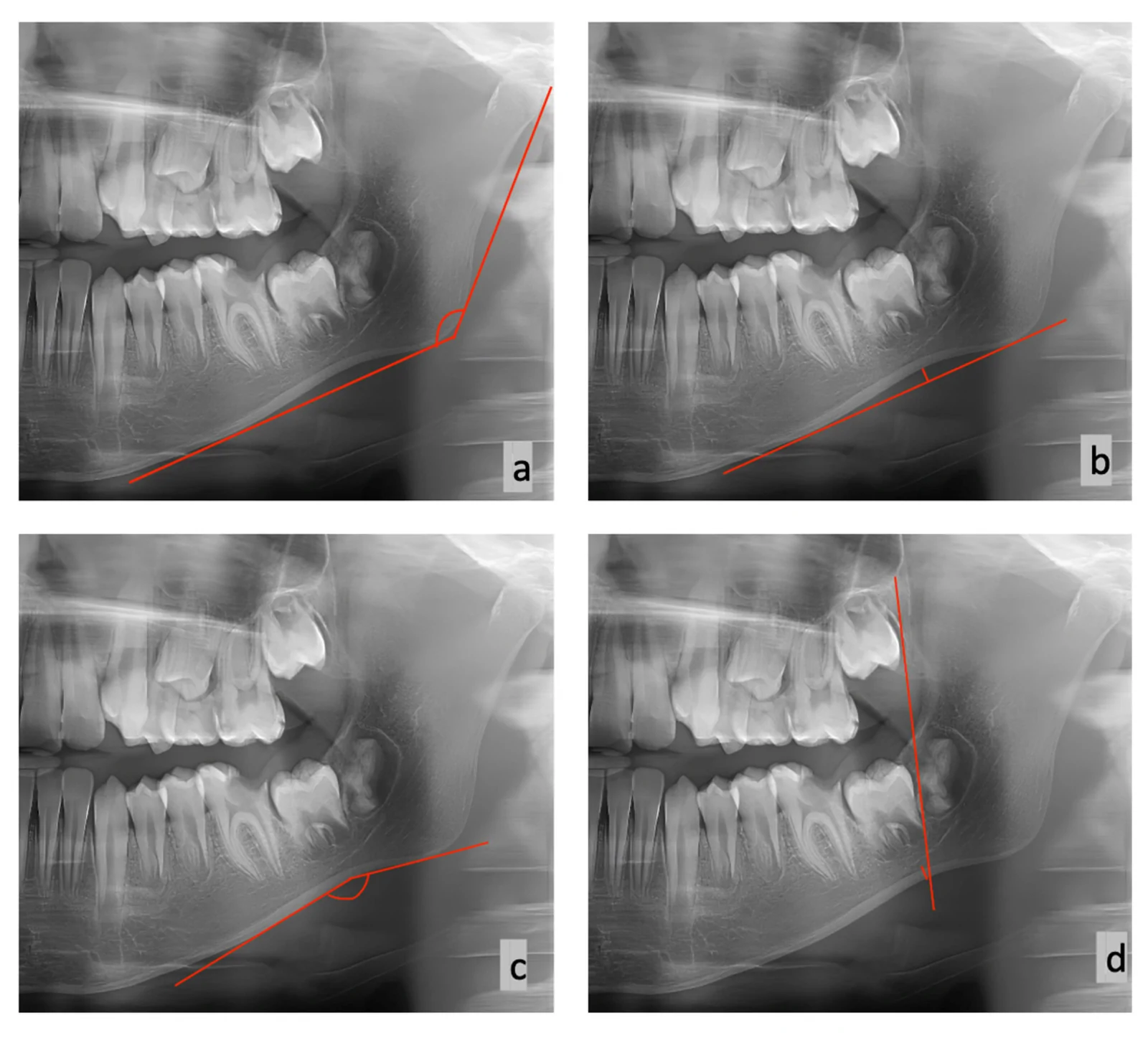
Radiomorphometric measurements of the mandible. (**a**) Gonial angle (GA): the angle formed between the tangent to the inferior border of the mandible and the tangent to the posterior border of the ramus. (**b**) Antegonial notch depth (AND): the perpendicular distance between the tangent to the inferior border of the mandible and the deepest point of the antegonial notch. (**c**) Antegonial notch angle (ANA): the angle formed by two lines extending from the deepest point of the antegonial notch to its anterior and posterior borders. (**d**) Antegonial cortical thickness (ACT): the cortical bone thickness measured perpendicular to the mandibular cortex at the point where the best-fit linear reference line along the anterior border of the ascending ramus intersects the inferior border of the mandible.

Morphological measurements were performed on the side of the mandible where early FPM extraction had been carried out. GA, AND, ANA, and ACT were measured as described by Erzurumlu et al. [13] (Figure 4).

### 2.6. Fractal Analysis

**Figure 5:**
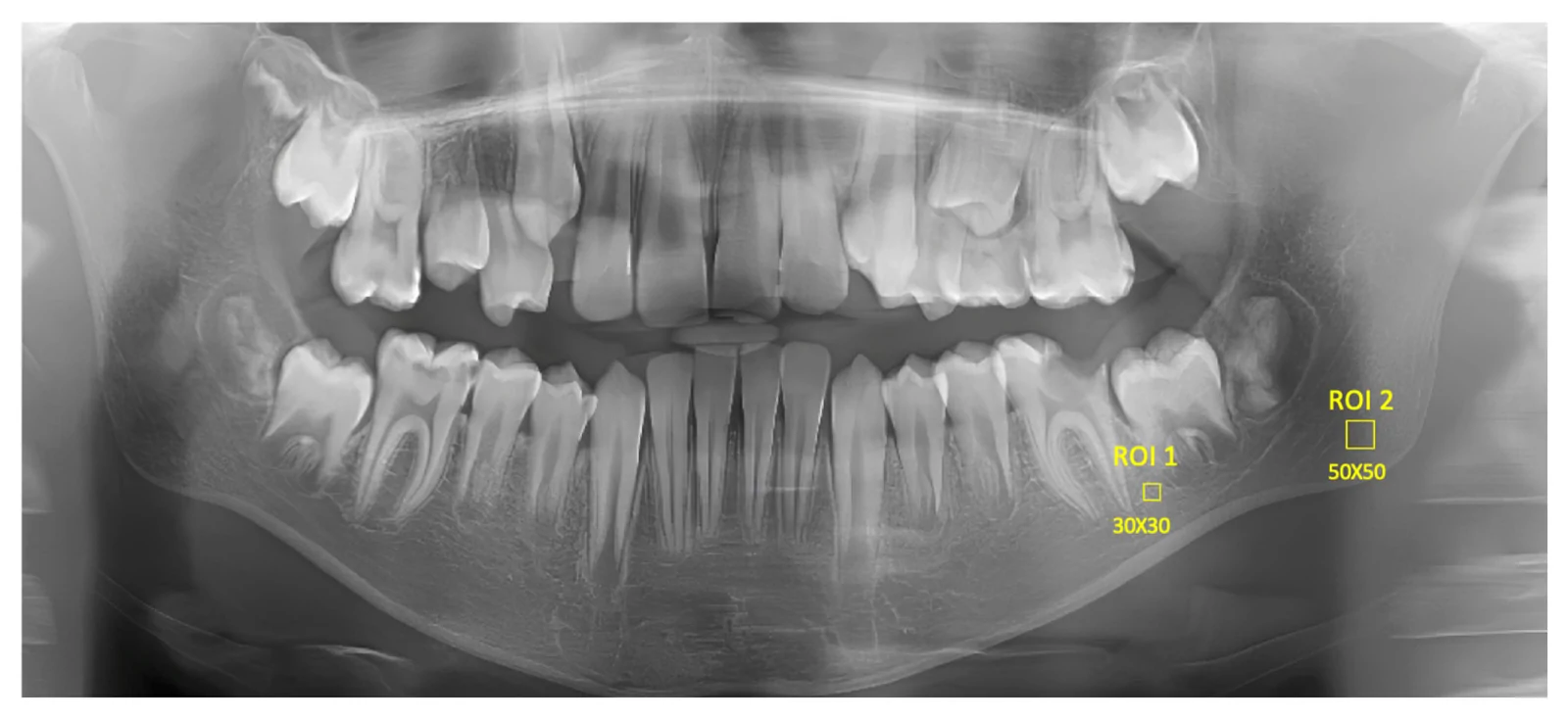
Regions of interest (ROIs) selected from the mandibular corpus and angulus.

DICOM panoramic images were analyzed using ImageJ 1.54 (National Institutes of Health, Bethesda, MD, USA). Two ROIs were selected: a 30 × 30-pixel region between the apical areas of the FPM and SPM in the mandibular corpus, and a 50 × 50-pixel region in the mandibular angulus. ROIs excluded tooth roots, periodontal spaces, the mandibular canal, and cortical bone. ROI dimensions were identical across patients, and no spatial resizing or resampling was performed (Figure 5).The nominal pixel size of the exported panoramic images was 28.4 µm. Accordingly, the 30 × 30-pixel and 50 × 50-pixel ROIs corresponded to nominal image dimensions of approximately 0.852 × 0.852 mm and 1.420 × 1.420 mm, respectively. No spatial resizing or resampling was performed before ROI selection (Figure 5).

**Figure 6:**
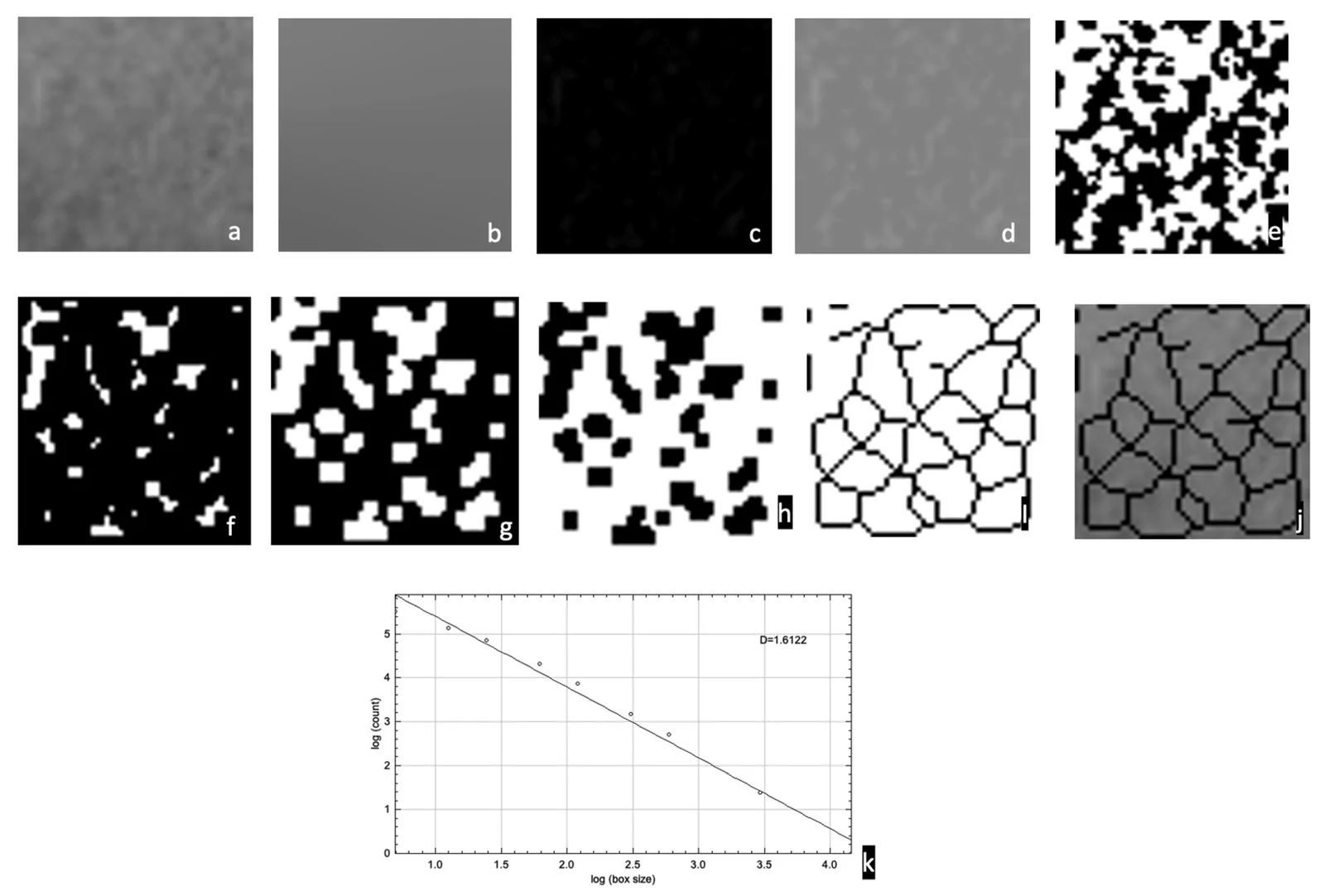
Image-processing steps for fractal analysis. (**a**) Original region of interest (ROI) selected from the panoramic radiograph. (**b**) Duplicated ROI after application of the Gaussian blur filter. (**c**) Image obtained by subtracting the blurred image from the original ROI. (**d**) Image obtained after adding a gray value of 128 to each pixel. (**e**) Binary image obtained after thresholding. (**f**) Image after erosion. (**g**) Image after dilation. (**h**) Image after inversion. (**i**) Skeletonized image representing the trabecular structure. (**j**) Superimposition of the skeletonized image onto the original ROI to verify its correspondence with the trabecular architecture. (**k**) Log–log regression plot used to calculate the fractal dimension (FD) using the box-counting method.

Image processing followed a protocol adapted from White and Rudolph [11]. Each ROI was converted to 8-bit grayscale and duplicated. The duplicate was blurred using a Gaussian filter (sigma = 10) [14], subtracted from the original, and a gray value of 128 was added. Images were binarized at a threshold of 128, eroded, dilated, inverted, and skeletonized. Skeletonized images were compared with the original ROIs to check correspondence with the trabecular pattern. FD was then calculated using box counting. Identical processing settings were applied to all images (Figure 6).

### 2.7. Statistical Analysis

Initial statistical analyses were performed using IBM SPSS Statistics (version 26.0; IBM Corp., Armonk, NY, USA). The normality of continuous variables was assessed using the Shapiro–Wilk test. Normally distributed continuous variables were summarized as mean ± standard deviation (SD), whereas non-normally distributed continuous variables and ordinal variables were presented as median (first–third quartiles, Q1–Q3). Categorical variables were summarized as frequencies and within-group percentages.

Age was compared using an independent-samples *t*-test. Normally distributed angular measurements were compared using Welch’s independent-samples *t*-test, whereas non-normally distributed continuous variables and ordinal variables were compared using the Mann–Whitney U test. Pearson’s chi-square test was used to compare sex distribution and the three-category PSM–SP status between the FO and UFO groups.

Bonferroni correction was applied to the six mandibular morphological and trabecular comparisons: GA, AND, ANA, ACT, corpus FD, and angulus FD. Adjusted *p*-values were calculated by multiplying the unrounded *p*-values by six, with values exceeding 1.000 capped at 1.000. The remaining comparisons were treated as exploratory and reported using unadjusted *p*-values.

An exploratory multivariable binary logistic regression model was fitted using maximum likelihood estimation to assess the association between AND and a FO after adjustment for the included covariates. FO was coded as the event. The model included AND, sex, initial SPM–mandibular plane angle, initial SP–mandibular plane angle, PAI score, and PSM–SP status. The latter was entered as a three-category variable, with PSM present as the reference category; male sex was the reference category for sex. All predictors were entered simultaneously. Continuous predictors and PAI score were entered as numeric terms, assuming linear associations with the log odds of a FO. Adjusted odds ratios (ORs), Wald-based 95% confidence intervals (CIs), and two-sided Wald *p* values were calculated. The overall association of PSM–SP status with outcome was assessed using a joint Wald test with two degrees of freedom. Overall model significance was assessed using the likelihood-ratio chi-square test against an intercept-only model, and Nagelkerke R^2^ was reported as a pseudo-R^2^ measure. Given the limited sample size relative to the number of estimated parameters, the multivariable findings were interpreted as exploratory.

Receiver operating characteristic (ROC) analysis was performed for AND, the only morphological or trabecular parameter showing a significant between-group difference after Bonferroni correction. FO was designated as the positive outcome, with higher AND values indicating a greater likelihood of FO. The area under the ROC curve (AUC) was calculated, and its 95% confidence interval (CI) was estimated using the DeLong method. The threshold maximizing the Youden index (sensitivity + specificity − 1) was identified, and the corresponding sensitivity and specificity were reported. Because the threshold was selected and evaluated in the same sample, it was considered exploratory and subject to external validation before clinical application.

All tests were two-sided. Statistical significance was defined as *p* < 0.05 for exploratory unadjusted comparisons and multivariable analyses, and as Bonferroni-adjusted *p* < 0.05 for the six morphological and trabecular comparisons.

## 3. Results

A total of 60 patients were included in the study, with 30 patients each in the FO and UFO groups. The mean age was 9.53 ± 1.27 years in the FO group and 9.94 ± 1.41 years in the UFO group (*p* = 0.238). No significant between-group differences were observed in sex distribution (*p* = 0.121), mandibular SPM developmental stage (*p* = 0.968), or TPM developmental stage (*p* = 0.260). Similarly, the initial SPM–mandibular plane angle (*p* = 0.952), initial SP–mandibular plane angle (*p* = 0.838), and PAI score (*p* = 0.717), did not differ significantly between the groups. However, the distribution of PSM-SP status at baseline differed significantly between the groups (*p* = 0.033). An extracted PSM with an unerupted SP was more frequent in the FO group (60.0%) than in the UFO group (26.7%) (Table 1).

Table 2 summarizes the between-group comparisons of the six mandibular morphological and trabecular parameters evaluated in this study. AND was significantly greater in the FO group than in the UFO group (unadjusted *p* = 0.002), and this difference remained significant after Bonferroni correction (adjusted *p* = 0.015). No significant between-group differences were observed in GA, ANA, ACT, or FD in either the corpus or angulus regions.

An exploratory multivariable binary logistic regression analysis was performed to assess whether AND was associated with a FO after adjustment for sex, initial SPM–mandibular plane angle, SP–mandibular plane angle, PAI score, and PSM-SP status. The overall model was statistically significant (likelihood-ratio χ^2^(7) = 18.50, *p* = 0.010; Nagelkerke R^2^ = 0.354). Each 1 mm increase in AND was associated with higher odds of a FO (adjusted OR = 2.22, 95% CI: 1.19–4.15; *p* = 0.012). The overall association of PSM–SP status with outcome was also statistically significant (joint Wald *p* = 0.021). Compared with cases in which the PSM was present, cases in which it had been extracted and the SP remained unerupted had higher odds of a FO (adjusted OR = 9.51, 95% CI: 1.45–62.50; *p* = 0.019). An erupted SP was not significantly associated with outcome (adjusted OR = 1.94, 95% CI: 0.28–13.57; *p* = 0.503). None of the remaining predictors showed a statistically significant association. The category-specific estimates should be interpreted cautiously because of the small subgroup sizes and wide confidence intervals (Table 3).

### ROC Analysis

**Figure 7:**
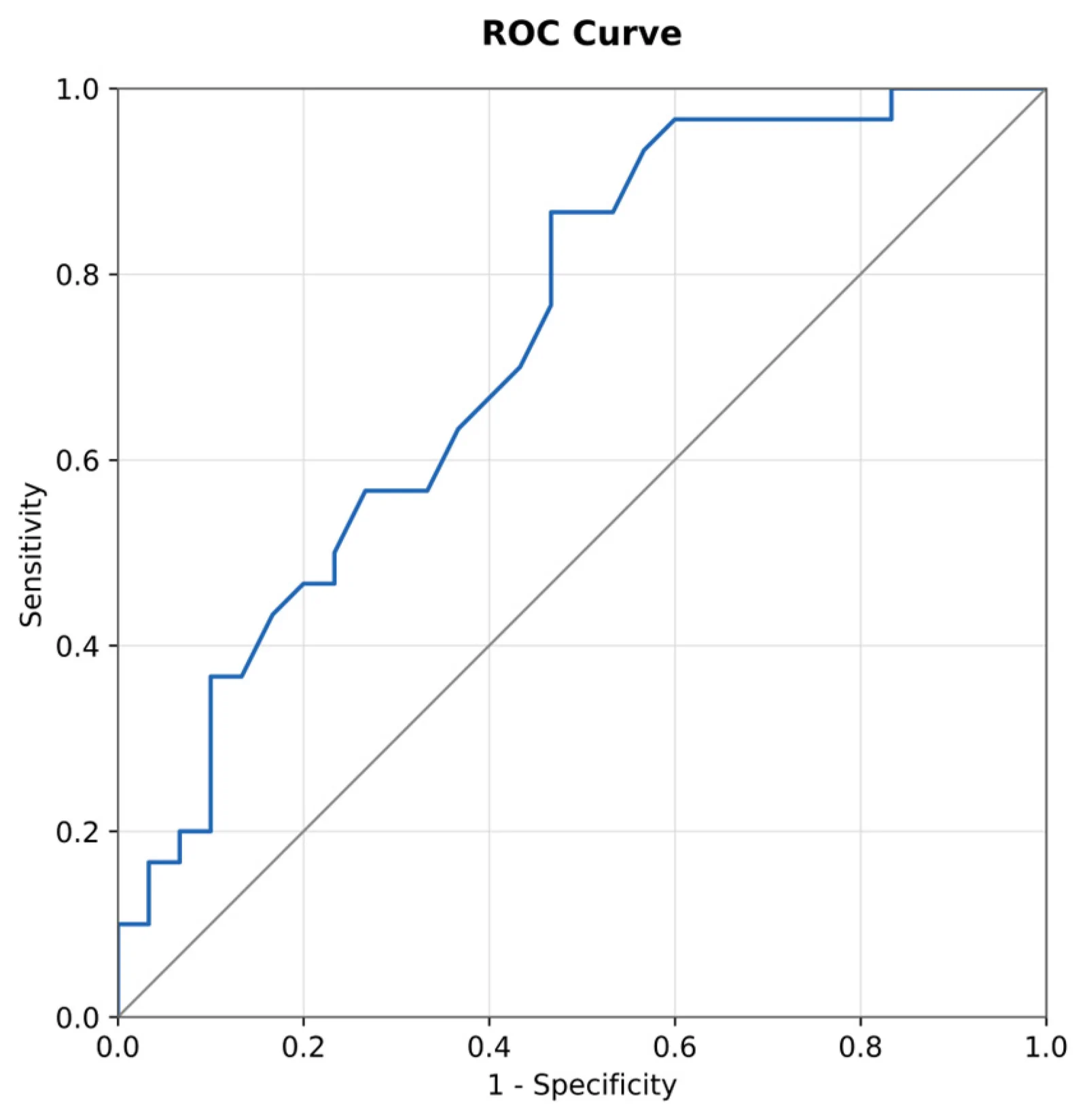
Receiver operating characteristic (ROC) curve for antegonial notch depth in discriminating favorable from unfavorable long-term outcomes following early mandibular first permanent molar extraction. The AUC was 0.728 (95% CI: 0.600–0.856, DeLong method). The maximum Youden index was 0.400. An AND threshold of ≥2.0 mm yielded a sensitivity of 86.7% and a specificity of 53.3%. The diagonal line indicates chance-level discrimination (AUC = 0.5). The threshold was derived and evaluated in the same cohort and should be interpreted as exploratory, pending independent validation.

As AND was the only one of the six mandibular morphological and trabecular parameters showing a significant between-group difference after Bonferroni correction, ROC analysis was performed to explore its ability to discriminate between FO and UFO. AND demonstrated moderate discrimination, with an AUC of 0.728 (95% CI: 0.600–0.856, calculated using the DeLong method). The maximum Youden index was 0.400; classifying AND values ≥ 2.0 mm as indicative of a FO yielded a sensitivity of 86.7% and a specificity of 53.3%. Given its relatively low specificity and its derivation and evaluation within the same cohort, the approximately 2.0 mm threshold should be regarded strictly as an exploratory benchmark rather than a clinical decision threshold. Independent validation is required before its clinical utility can be established (Figure 7).

## 4. Discussion

The principal finding of this study was that greater pre-extraction AND was associated with favorable long-term outcomes following early mandibular FPM extraction. The between-group difference remained significant after Bonferroni correction, and the association persisted after adjustment for the included covariates. AND also demonstrated moderate discrimination between outcome groups. Baseline PSM–SP status was associated with outcome in the exploratory regression model, whereas initial tooth angulations, PAI score, and sex were not. The morphological and trabecular null hypothesis was therefore rejected on the basis of AND, and the additional dental and periapical null hypothesis on the basis of PSM–SP status. Nonsignificant findings for GA, ANA, ACT, and FD remain inconclusive given the limited sample size and correction for multiple comparisons.

Favorable SPM migration and spontaneous space closure following FPM extraction remain unpredictable, particularly in the mandible [5]. Although systematic reviews have identified age, SPM developmental stage and angulation, and TPM presence as potential prognostic factors, reliable prediction remains limited by predominantly retrospective evidence of low certainty [3,5]. More recent studies have further suggested that spontaneous space closure may not depend solely on extraction timing and dental developmental stage. In addition to dental factors such as the initial angulation of the SPM, the position of the SP, and the presence of the TPM, mandibular morphological characteristics may also be associated with long-term outcomes [4,6].

Accordingly, the present study investigated the relationship between long-term spontaneous space closure and mandibular morphological and trabecular bone characteristics, which have received relatively limited attention in the existing literature. In addition, potential dental and local factors were recorded, including the initial angulations of the adjacent SPM and SP, the presence of the PSM or the eruption status of its permanent successor and the PAI score of the extracted FPM. An important methodological feature of the present study was that the FO and UFO groups were comparable in terms of chronological age and the developmental stage of the mandibular SPM, with no significant difference in sex distribution between the groups. Given that age and SPM developmental stage are considered important factors associated with spontaneous space closure, comparability between the groups for these characteristics may have reduced potential confounding related to developmental differences.

Sex has been investigated as a potential factor associated with favorable spontaneous space closure following FPM extraction. Papaioannou et al. reported no significant association between sex and successful space closure. Although upright SPM positioning differed between males and females in their unadjusted analysis, sex was not a significant predictor in the regression model [15]. Similarly, in the present study, sex was not significantly associated with FO after adjustment for the other included variables.

Pre-extraction angulation of the SPM and SP has been investigated as a potential factor associated with spontaneous space closure following early extraction of the FPM, using different reference lines across studies. In the present study, neither the baseline SPM–mandibular plane angle nor the SP–mandibular plane angle differed significantly between the FO and UFO groups. This finding is consistent with that of Celik et al., who reported no significant association between SPM angulation and successful spontaneous space closure in the mandibular arch [4]. Similarly, Aldahool et al. assessed SPM and SP angulation relative to the long axis of the FPM but did not confirm a significant association between initial mesial SPM angulation and successful spontaneous space closure Nordeen et al. found no significant association using the occlusal plane as the angular reference. Their evaluation of a prediction model incorporating SPM angulation and TPM presence showed no additional predictive value over age or SPM developmental stage [16].

In contrast, Patel et al. reported a significant association between mesial SPM angulation and successful spontaneous space closure in the mandibular arch [17]. Teo et al. also emphasized the potential predictive value of mesial SPM angulation when considered alongside TPM presence and SPM developmental stage [6]. These findings highlight the potential importance of considering initial angulation alongside other developmental characteristics. Discrepancies across studies may reflect differences in the reference lines used for angular measurements, the treatment of angulation as a continuous or categorical variable, the age and dental developmental profiles of the study populations, and the criteria used to define successful space closure. Moreover, the FO definition in the present study incorporated both space closure and an acceptable axial position of the SPM. This distinction should be considered when comparing our findings with studies that primarily assessed space closure.

Teo et al. evaluated the position of the SP within the furcation of the PSM as a potential radiographic predictor of spontaneous space closure following mandibular FPM extraction [6]. Aldahool et al. also investigated this relationship but did not confirm a significant association with successful spontaneous space closure [7]. In the present study, PSM–SP status was classified into three categories: PSM present, PSM extracted with the SP unerupted, and SP erupted. Cases in which the PSM had been extracted and the SP remained unerupted were more frequent in the FO group. In the multivariable analysis, this category was associated with significantly higher odds of a FO than the reference category of a retained PSM. Mesial tipping of the FPM was observed on pre-extraction radiographs in most of these cases; however, previous records were unavailable, preventing quantification of positional changes. Mesial migration of the FPM and subsequent local space loss may occur following premature loss of the PSM, particularly in the absence of space maintenance [18]. Such changes may alter the spatial relationships at the extraction site and potentially influence the subsequent eruption and positioning of the SPM. Nevertheless, because pre-extraction mesial migration and its progression were not quantitatively assessed, this proposed explanation remains an observational hypothesis.

Another potential predictor evaluated in this study was the pre-extraction PAI score of the FPM. The PAI is an ordinal scoring system that reflects the radiographic severity of apical periodontitis, with higher scores indicating more pronounced periapical bone changes rather than directly measuring lesion size [19]. A prospective study reported accelerated eruption of permanent successor premolars following the extraction of infected primary molars, accompanied by a greater frequency of absent overlying alveolar bone [20]. Although this evidence concerns a different anatomical relationship, it provided a rationale for hypothesizing that more extensive periapical bone changes associated with higher FPM PAI scores might facilitate mesial movement of the SPM following extraction. However, PAI scores were not significantly associated with FO in the present study. Because SPM movement was not directly quantified, these findings do not establish whether the initial periapical condition influenced the extent or rate of mesial movement. Specifically, cases in which the PSM had been extracted and the SP remained unerupted showed higher adjusted odds of a FO than cases in which the PSM was present.

In the present study, several mandibular morphological characteristics that may be associated with the eruption position and spontaneous mesial migration of the SPM following mandibular FPM extraction were evaluated, including the GA, AND, ANA, and ACT in the antegonial region. The GA is a radiographic morphological parameter used to characterize mandibular configuration, and differences in panoramic GA measurements have been reported among adults with different mandibular divergence patterns [9]. In the present study, the GA did not differ significantly between the FO and UFO groups. This finding is consistent with that of Celik et al., who evaluated five-year outcomes following FPM extraction [4]. In contrast, Celik et al. reported a significant association between the ANA and spontaneous space closure. In the present study, however, the ANA did not differ significantly between the groups, whereas the AND was significantly greater in the FO group. This discrepancy may be attributable to the fact that ANA and AND represent distinct geometric characteristics of the same anatomical region. Thus, although the present findings suggest an association between AND and favorable long-term outcomes, they do not establish the biological mechanism underlying this relationship.

Previous longitudinal studies have linked deeper antegonial notches to more vertically directed mandibular growth and smaller increases in mandibular corpus length [21,22]. Growth and remodeling patterns reflected by notch morphology could influence the spatial relationships relevant to SPM eruption and subsequent space closure. In particular, a smaller increase in mandibular corpus length might limit anteroposterior changes in the region of the extraction site. However, this remains an untested hypothesis, and vertically directed growth cannot be assumed to facilitate SPM mesial migration. Kolodziej et al. found notch depth to be a weak predictor of subsequent mandibular growth [23]. Moreover, the classical implant studies of Björk and Skieller documented mesial migration of the mandibular dentition in association with forward mandibular rotation, while emphasizing substantial individual variability [24]. The greater AND in the FO group may therefore reflect anatomical or developmental characteristics associated with outcome without directly identifying the mechanism of space closure. Because growth rotation and post-extraction tooth movement were not assessed longitudinally, the present study cannot distinguish between these explanations.

ROC analysis demonstrated moderate discrimination between outcome groups (AUC = 0.728; 95% CI: 0.600–0.856). A threshold of approximately 2.0 mm yielded high sensitivity (86.7%) but limited specificity (53.3%), suggesting potential for AND as a complementary radiomorphometric indicator. However, notch depth has limited value for predicting subsequent mandibular growth and may change during growth [23,25]. Its association with space-closure outcomes should therefore be examined across different ages at FPM extraction. Because the threshold was selected and evaluated in the same cohort, its apparent performance may be optimistic. It should be regarded as an exploratory benchmark rather than a clinical decision threshold, pending independent validation.

No significant difference in cortical bone thickness in the antegonial region was observed between the FO and UFO groups. Mandibular cortical thickness has previously been reported to vary with age, dental maturation, and craniofacial growth characteristics and may serve as a radiomorphometric parameter for assessing the growing mandible [26]. In the present study, however, the comparability of the groups in terms of age, sex, and SPM developmental stage may have minimized the influence of these developmental factors on ACT, which may partly explain the absence of a significant difference.

FD is a quantitative parameter used to characterize the structural complexity of radiographic trabecular bone patterns and has been widely applied in dentistry for the assessment of trabecular bone organization [12,14,27]. Although FD has been investigated as a potential imaging biomarker for osteoporosis screening, the available evidence remains limited in certainty, and FD should not be interpreted as a direct measure of bone mineral density [28]. In the present study, baseline trabecular organization was hypothesized to be associated with long-term spontaneous space closure following mandibular FPM extraction. Accordingly, FA was performed on pre-extraction panoramic radiographs to investigate this potential association.

FD values derived from FA may vary depending on the anatomical region evaluated and the image-processing protocol used [27,29]. Therefore, in the present study, ROIs were selected from two mandibular regions, the corpus and angulus, to evaluate trabecular bone complexity in relation to the developing SPM. No significant differences in FD values obtained from either the mandibular corpus adjacent to the extraction site or the mandibular angulus were observed according to long-term outcome. Considering that the FO and UFO groups were also comparable in terms of age, mandibular SPM developmental stage, and sex, these findings suggest that baseline radiographic trabecular complexity alone may not be a major factor associated with long-term spontaneous mesial migration of the mandibular SPM and subsequent space closure.

Tooth eruption and spontaneous tooth movement involve a dynamic remodeling process characterized by coordinated bone resorption and formation within the surrounding alveolar bone [30]. However, FD derived from panoramic radiographs does not directly measure this biological activity; rather, it reflects the two-dimensional geometric complexity of the trabecular bone pattern at the time of image acquisition [12]. Therefore, the similar pre-extraction FD values observed in the FO and UFO groups in the present study do not necessarily imply that alveolar bone remodeling following mandibular FPM extraction, or subsequent changes in trabecular organization over time, were also similar between the two groups. Indeed, previous study have demonstrated that time-dependent changes in trabecular organization following tooth extraction can be radiographically monitored using FA [31]. Accordingly, longitudinal assessment of FD changes on follow-up radiographs until the SPM reaches its occlusal position may provide further insight into the potential role of trabecular bone dynamics in spontaneous mesial migration of the SPM.

Identifying reproducible radiographic predictors of favorable spontaneous space closure may provide a foundation for future AI-assisted prognostic models. Once validated in larger, independent cohorts, morphological and dental features investigated in studies such as the present one could be incorporated into models designed to estimate outcomes following early mandibular FPM extraction. Recent work by Chen et al. evaluating a vision-language model for detecting condylar osseous changes on CBCT illustrates the potential of AI-assisted dental image interpretation [32]. However, applying such approaches to spontaneous space closure would require task-specific development and external validation before clinical use.

Several limitations should be acknowledged. The retrospective, single-center design and modest sample size limit generalizability and introduce potential selection bias. The sample-size calculation did not account for Bonferroni correction; therefore, nonsignificant findings for GA, ANA, ACT, and FD remain inconclusive. The multivariable findings and ROC estimates are exploratory. The 2.0-mm AND threshold was selected and evaluated in the same cohort and showed limited specificity (53.3%), requiring independent validation before clinical use. Incomplete documentation of extraction indications and the timing of other FPM extractions precluded adjustment for these factors; PAI scores captured only periapical status. Panoramic projection geometry, regional magnification, patient positioning, and anatomical superimposition may introduce measurement error or systematic bias in AND and FD assessments. FD is also sensitive to image resolution, ROI selection, and processing. The original assessments were not blinded to outcome, and subsequent blinded reliability testing could not eliminate potential bias in those assessments. The absence of longitudinal measurements precluded evaluation of growth, tooth movement, and bone remodeling over time. Larger multicenter studies with blinded assessments and longitudinal follow-up are needed to validate these findings.

## 5. Conclusions

Greater pre-extraction AND was associated with favorable long-term outcomes following early mandibular FPM extraction, and this association remained statistically significant after adjustment for the included covariates. PSM-SP status was also associated with outcome in the exploratory multivariable analysis. No statistically significant between-group differences were detected in GA, ANA, ACT, or trabecular FD; however, these findings remain inconclusive given the limited sample size and correction for multiple comparisons. The potential prognostic relevance of AND and the additional dental findings requires confirmation in larger, prospective cohorts before clinical application.

## Figures and Tables

**Table 1:** Baseline demographic, developmental, and potential dental factors according to the final outcome group.

Characteristic	FO Group (*n* = 30)	UFO Group (*n* = 30)	*p* Value
Age (years), mean ± SD	9.53 ± 1.27	9.94 ± 1.41	0.238 ^a^
SPM developmental stage, median (Q1–Q3)	5 (5–6)	5 (5–6)	0.968 ^b^
TPM developmental stage, median (Q1–Q3)	1.5 (1–2)	1 (0–2)	0.260 ^b^
Sex, n (male/female)	19/11	13/17	0.121 ^c^
Initial SPM–mandibular plane angle (°), mean ± SD	83.10 ± 7.31	83.23 ± 9.77	0.952 ^a^
Initial SP–mandibular plane angle (°), mean ± SD	98.94 ± 11.41	99.50 ± 9.38	0.838 ^a^
PAI score, median (IQR)	2.0 (2.0–3.75)	2.0 (2.0–5.0)	0.717 ^b^
PSM–SP status at baseline, n (%)			**0.033 ^c^**
PSM present	4 (13.3)	7 (23.3)	
PSM extracted; SP unerupted	18 (60.0)	8 (26.7)	
SP erupted	8 (26.7)	15 (50.0)	

^a^ Independent-samples *t*-test for age and Welch’s *t*-test for angular measurements; ^b^ Mann–Whitney U test; ^c^ Pearson’s chi-square test. Dental developmental stages were numerically coded for statistical analysis (Demirjian stages A–H = 1–8, respectively); for third molars, 0 denoted the germ stage preceding stage A. Data are presented as mean ± SD, median (Q1–Q3), or n (%), unless otherwise indicated. Percentages were calculated within each outcome group. Statistically significant values are shown in bold. FO, favorable outcome; UFO, unfavorable outcome; SD, standard deviation; Q1, first quartile; Q3, third quartile; SPM, second permanent molar; TPM, third permanent molar; PSM, primary second molar; SP, second premolar; PAI, periapical index.

**Table 2:** Comparison of radiomorphometric and fractal parameters between the favorable and unfavorable outcome groups.

Variable	FO (*n* = 30)	UFO (*n* = 30)	*p* Value	Bonferroni-Adjusted *p*
Gonial angle (°)	130.36 ± 5.34	129.02 ± 6.07	0.369 ^a^	1.000
Antegonial notch depth (mm)	2.65 (2.20–3.85)	1.90 (1.45–2.58)	**0.002 ^b^**	**0.015**
Antegonial notch angle (°)	159.13 ± 5.97	161.70 ± 6.13	0.105 ^a^	0.633
Antegonial cortical thickness (mm)	2.05 (1.73–3.88)	2.20 (1.93–2.60)	0.700 ^b^	1.000
Corpus fractal dimension	1.37 (1.31–1.39)	1.37 (1.32–1.41)	0.483 ^b^	1.000
Angulus fractal dimension	1.49 (1.43–1.53)	1.50 (1.47–1.54)	0.204 ^b^	1.000

^a^ Welch’s independent-samples *t*-test; ^b^ Mann–Whitney U test. Data are presented as mean ± SD or median (Q1–Q3), as appropriate. Bonferroni-adjusted *p*-values were calculated by multiplying the unrounded *p*-values by six; values exceeding 1.000 were capped at 1.000. Bold values indicate comparisons with Bonferroni-adjusted *p* < 0.05. Statistically significant values are shown in bold. FO, favorable outcome; UFO, unfavorable outcome; SD, standard deviation; Q1, first quartile; Q3, third quartile.

**Table 3:** Multivariable logistic regression analysis of factors associated with a favorable outcome.

Predictor	Adjusted OR	95% CI	*p* Value
Antegonial notch depth, per 1 mm increase	**2.22**	**1.19–4.15**	**0.012**
Female sex	0.40	0.11–1.44	0.162
Initial SPM–mandibular plane angle, per 1° increase	1.01	0.94–1.09	0.692
Initial SP–mandibular plane angle, per 1° increase	1.00	0.94–1.06	0.972
PAI score, per one-point increase	1.03	0.65–1.64	0.889
PSM–SP status (overall)	—	—	**0.021**
PSM present	Reference	—	—
PSM extracted; SP unerupted	**9.51**	**1.45–62.50**	**0.019**
SP erupted	1.94	0.28–13.57	0.503

Favorable outcome was coded as the event. All predictors were entered simultaneously into the model. Reference categories were male sex and primary second molar present. PAI score was entered as a numeric predictor, assuming a linear association with the log odds of a favorable outcome. Predictor-specific *p* values were obtained using Wald tests; the overall *p* value for primary second molar–second premolar status was obtained using a joint Wald test with two degrees of freedom. Bold values indicate *p* < 0.05. Estimates should be interpreted as exploratory because of the limited sample size relative to the number of model parameters and the wide confidence intervals. Statistically significant values are shown in bold. OR, odds ratio; CI, confidence interval; SPM, second permanent molar; SP, second premolar; PSM, primary second molar; PAI, periapical index.

## Data Availability

The data supporting the findings of this study are available from the corresponding author upon reasonable request.

## Abbreviations

**ACT**	Antegonial cortical thickness
**ANA**	Antegonial notch angle
**AND**	Antegonial notch depth
**AUC**	Area under the curve
**CI**	Confidence interval
**FA**	Fractal analysis
**FD**	Fractal dimension
**FO**	Favorable outcome
**FPM**	First permanent molar
**GA**	Gonial angle
**IQR**	Interquartile range
**OR**	Odds ratio
**ROC**	Receiver operating characteristic
**ROI**	Region of interest
**SD**	Standard deviation
**SP**	Second premolar
**SPM**	Second permanent molar
**TPM**	Third permanent molar
**UFO**	Unfavorable outcome
**PSM**	Primary second molar
